# Trauma-related retrobulbar hematoma: predictive risk factors and clinical outcome in 52 cases

**DOI:** 10.1007/s00068-026-03296-0

**Published:** 2026-08-18

**Authors:** Ákos Bicsák, Hüseyin Şahin, Lena Brandt, Stefan Hassfeld, Lars Bonitz

**Affiliations:** 1 Health Faculty, University of Witten/Herdecke, Alfred-Herrhausen-Strasse 45, D-58453 Witten, Germany; 2 Clinic for Cranial- and Maxillofacial Surgery, Regional Plastic Surgery, Dortmund General Hospital, Chair of the University of Witten-Herdecke, Muensterstrasse 240, D-44145 Dortmund, Germany

**Keywords:** Orbital trauma, Retrobulbar hematoma, Orbital compartment syndrome, Outcome, Predictor

## Abstract

**Purpose:**

Retrobulbar hematoma (RBH) is a vision-threatening emergency. This large-scale study integrates clinical data and 3D analysis to identify prognostic factors, refine diagnosis, and support guideline-based emergency management.

**Methods:**

This study standardizes terminology, applies AO fracture classification, and uses CT-based 3D segmentation to analyze anatomical patterns, treatment protocols, providing evidence-based guidance for diagnosis, surgical management, and postoperative care.

**Results:**

The Dortmund Maxillofacial Trauma Registry (2018–2025) recorded 52 RBHs among 7,671 trauma patients (0.7%). RBH occurred predominantly in middle-aged males and elderly females, with overproportionate incidence of anticoagulant therapy. Most fractures clustered around the orbital floor, medial wall, zygomatic surface, often near the Frankfurt horizontal plane. RBHs were mainly extraconal; poor outcome was predicted with intraconal location, optic nerve contact, orbital floor fracture, high RBH/orbital volume ratio, and aspirin use. Surgical decompression was performed in 15 cases (28.9%); 65,4% (34 of 52) retained vision. Neural network analysis confirmed fracture site and hematoma position as key prognostic factors over demographic or comorbidity variables.

**Conclusion:**

In 7,671 midface fracture cases, RBHs occurred in 0.7% (*n* = 52), mainly after trauma. Falls predominated in aetiology; orbital floor and zygomatic fractures were most frequent. Blindness occurred in 3.8%. Prompt decompression remains critical; volumetric analysis offers new prognostic insight. All midface fracture or periorbital hematoma patients require urgent ophthalmologic assessment with prompt decompression when indicated. A suggested checklist to support emergency diagnostics presents findings. We recommend 48-hour observation to prevent vision loss from delayed post-traumatic ocular complications.

## Introduction

Retrobulbar hematomas (RBH, also referred to as orbital hematoma or retrobulbar hemorrhage) can occur due to trauma, but may also develop for other reasons, such as postoperative complications after periorbital, orbital or ophthalmic surgeries [1], as adverse effects of medications or medical procedures, following episodes of vomiting or in individuals with hereditary coagulation disorders such as hemophilia or von Willebrand’s disease [2]. In cases of interpersonal violence (IPV) up to 80% present with orbital blunt trauma, more commonly on the left side due to predominantly right-handed assault [3].

The most important clinical facts regarding RBH are well established in the literature: it is an emergency situation that can lead to permanent loss of vision [2, 4, 5]. The anatomical localization, volume, association with other injuries, and the effect of different health-related factors on patient outcome have been thoroughly studied. The current literature contains many case reports and case series with outcome analysis [5–14], but far fewer studies provide in-depth clinical research into the background of the RBH or orbital compartment syndrome [4, 7, 15–18] (OCS, also see below).

Medical training varies between countries and RBH is not always adequately included in training programs [5, 14, 19]. Nearly two-thirds of emergency department physicians in the United Kingdom have never encountered an RBH [19]. A similar situation exists in other countries, some of which lack relevant literature altogether [5]. Therefore, such materials should be implemented in national guidelines and used to support training of emergency department team members.

The goal of this study was not only to analyze RBHs and the causes leading to blindness, but also to provide a checklist to facilitate early recognition of RBH and expedite therapeutic decision-making.

## Materials and methods

This study (No. 152/2017) was approved by the Ethics Commission of the University of Witten—Herdecke. This study was conducted in accordance with the Declaration of Helsinki, the laws and regulations of the European Union, the Federal Republic of Germany, the State of North-Rhine-Westfalia, and the General Hospital Dortmund.

A) Definitions, terminology
  The terminology of orbital hemorrhages is inconsistent in the literature. Below, we summarize the definitions found in the literature and indicate as how they served as a basis for the assessment of our study cases.
  **Orbital hematoma**: hemorrhage in the orbit (rarely used, anatomical localization not well-defined).
  **Retrobulbar hematoma (RBH)**:he most commonly used expression in the literature. Literally, the hematoma is located posterior to the bulb. It is most often a space-occupying process that leads to orbital compartment syndrome (see below). Many authors use the term synonimously with orbital compartment syndrome [20–23].
  **Subperiosteal hematoma**: term used in few papers [24], referring hematomas exclusively between the bony orbital wall and the periorbita; also a space-occupying process.
  **Intraconal hematoma**: retrobulbar hematoma within the cone formed by the straight eye muscles [25, 26].
  **Extraconal hematoma**: orbital hematoma outside of the space bounded by the bulb and the rectus muscles [25, 26].
  **Extraorbitalhematoma**: Hematoma in the periorbital or lid region outside the orbital volume defined by the bony orbital walls and the tarsal plates of the lids; less likely to lead to orbital compartement syndrome.
  **Orbital compartment syndrome (OCS)** or **acute orbital compartment syndrome (AOCS)**: it presents with well-defined symptoms and signs including proptosis / exophthalmus, severe pain with sudden onset, swelling, chemosis, subconjunctival hemorrhage, ophthalmoplegia, deterioration in visual acuity [20], deficient pupillary light reflex (PLR) [18] and reactive afferent pupillary defect (RAPD) [7, 23, 27, 28]. Some studies suggest that three out of four RBHs result in OCS [5]. The same study [5] has found an RBH in all OCS cases. A surgical decompression should be performed as soon as possible [7, 20].
  Hemorrhage and hematoma are both used interchangeably [29].

B) Classifications
**RBH classification**
  Zimmerer et al. presented a clinical classification with therapeutic recommendations [4]. Three severity grades (RBH I-II-III) were introduced: RBH with just radiologic signs or non-specific symptoms, RBH with symptoms or radiologic signs and RBH with symptoms and radiologic signs. Conservative therapy is suggested for the mild form and combined surgical and medical therapy for the two most severe forms (RBH II and III). This classification has not been validated for outcome predictions.
  This algorithm corresponds to our procedure, however, RBH II and III are primarily time-dependent categories in the emergency department, as CT-scaning is mandatory for patients with head and neck trauma [30].
**Fracture classification**
  The further fracture classification followed the current AO classification system [31].

C) Clinical procedure
  Our Department is integrated into the Trauma Centre of the Hospital and is responsible for the management of RBHs – in cooperation with other disciplines, including traumatology, neurosurgery, ophthalmology, radiology and anesthesiology and intensive care [30, 31]. Upon arrival of a patient with visual impairment after trauma at the Emergency Room, an immediate examination is performed. If a RBH is diagnosed clinically or radiologically, surgical decompression is performed immediately (unless the hematoma is older than 24 h) and further measures are initiated simultaneously.
  Daily follow-up examinations by ophthalmologists and maxillofacial surgeons are carried out.
  As supportive therapy, 500 mg – 1000 mg of intravenous methyl-prednisolone is administered perioperatively and repeated once within 24 h. The further need of corticosteroid therapy is evaluated together with the eye specialists. Antibiotics (ampicillin-sulbactam 3000 mg intravenously three times daily or equivalent) are administered for at least three days to prevent orbital infections. Adequate pain therapy with novaminsulfon (1000 mg three to four times daily) and cryotherapy are also provided.
  Any fractures of the orbit or periorbital bone are addressed only after vision is stabilized or the visus loss is considered permanent (usually after 3–5 days).

D) Surgical technique
  Most authors suggest lateral chantotomy with chantolysis [2, 4, 5, 14, 26, 32, 33]. Endoscopic decompression surgery is described in the literature [25], but we do not support this approach, as it requires special equipment, training and mostly more time to prepare, the post-operative observation is complicated and the resulting bony defect can increase the risk of orbital infections from the paranasal sinuses. Some authors report recovery even after delayed decompression [18, 33], therefore, up to 48 h post-trauma, the surgical intervention can be offered to the patients.
  The preferred method in our department is lateral canthotomy with infraorbital decompression [4]. This provides a good access to all quadrants, prevents further injuries to fine orbital structures and enables a good approach to potential bleeding sources. A soft rubber drain is placed in each wound.
  Fractures that need an open reduction and internal fixation are addressed in a second surgery after the swelling subsides. This enables a thorough revision of the orbit, a primary closure above the osteosynthesis implants and protects against secondary infections.

E) Post-hospital follow-up.
  The follow-up after discharge was individualized. Most patients are asked to present for at least one follow-up visit. If no complications arose, follow-up could be performed by external specialists at the patient’s request.

F) Study procedures
  During data analysis we considered facial fractures, orbital fractures, other associated injuries, general health conditions, and concomitant medication. Further details regarding our treatment protocol were also recorded.

### Classification of fractures

This study included patients with fractures of the head and neck region, who were treated in our department from 01.01.2007 to 30.09.2023. We used the current AO classification (AO Foundation, Davos, Switzerland) [34–38] of facial fractures and orbital fractures. We needed to add one more injury type: “fracture of the anterior wall of the maxillary sinus”. This entity was presented in our prior publications [39–41].

### Details of the retrobulbar hematoma

All patients with CT scan were included in the study. The acquisition of the CT-scans was performed at different hospitals, generally with a slice thickness of 1.5 mm or less. Further details to the CT devices cannot be provided. All scans were stored in the hospital PACS repository.

**Fig. 1:**
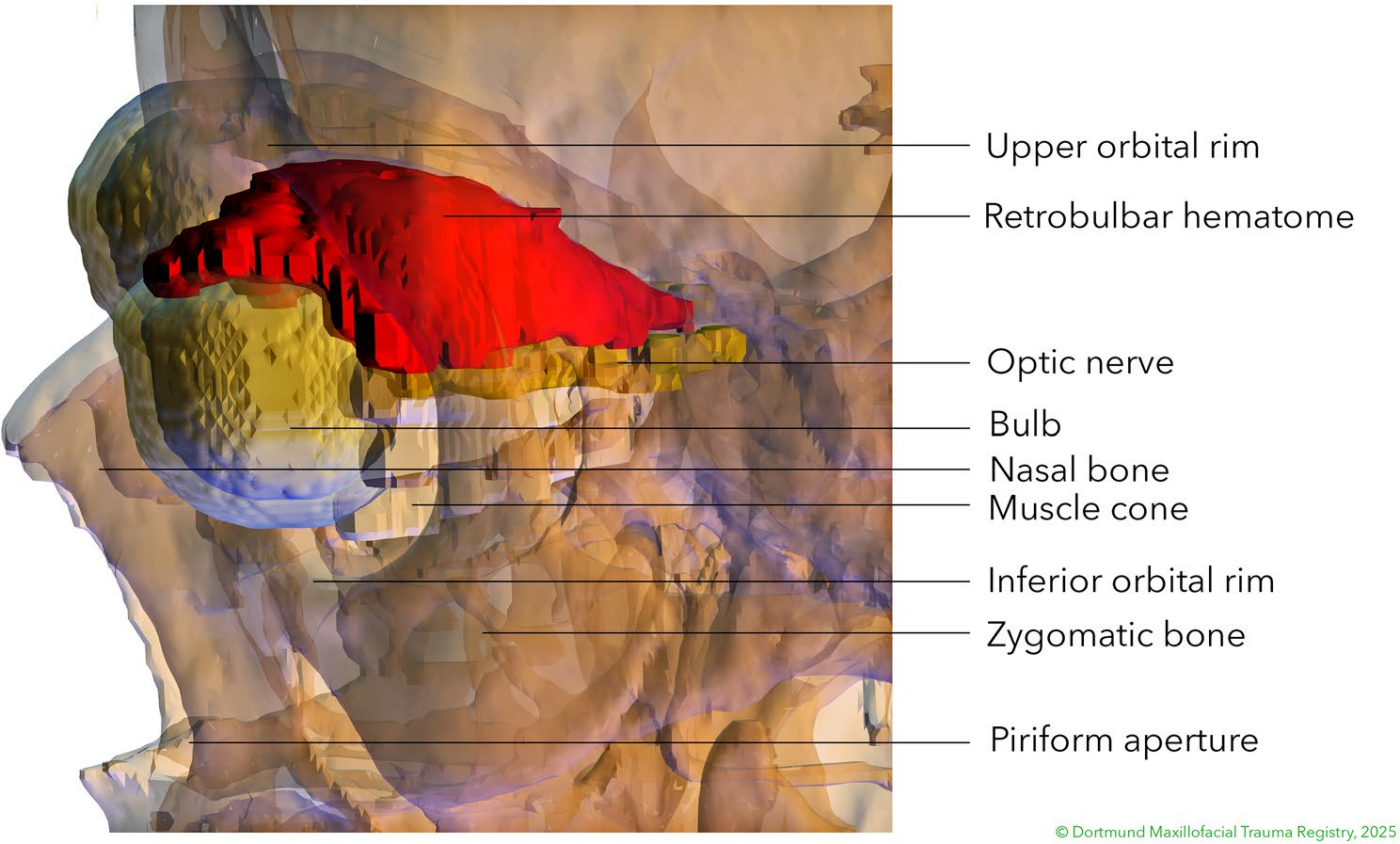
Presentation of the three-dimensional evaluation of the RBH: After segmentation, dimensions were measured with the software tools (BrainLab). The actual RBH presents an extraconal RBH in quadrants 1–2 (close contact to the bulb and to the optic nerve

The orbita was divided into four quadrants [42]: the upper quadrant was named as quadrant 1, the medial as quadrant 2, the inferior as quadrant 3 and the lateral as quadrant 4. The hematoma was segmented in the radiological image (Fig. 1) and its extent into the quadrants was examined.

The analysis of the scans was performed using the CMF Module of the Brainlab^®^ Origin Server^®^ (Brainlab SE, Munich, Germany; Version No. 3.4), a validated surgical planning and intraoperative navigation software. Scans were loaded with the software and bony structures and orbital content were auto-segmented and manually corrected, if needed. Hematoma in the orbit were segmented manually. Necessary measurements (distance, volume) were performed using the software measurement tools and calculation algorithms without further manual adjustment. Figure 1 presents the 3D evalutation of the RBH.

### Databank, statistics

We created the Dortmund Maxillofacial Trauma Registry, containing data of approximately 15,000 patients with head and neck injuries since 2007. The pseudonymized data were collected in the RedCap electronic data capture system hosted by Dortmund General Hospital. This web-based application provides secure data capture, an audit trail, and seamless integration with statistical programs [43, 44]. The further RBH analysis was performed after selecting the potential patients in the databank.

The measurements of anatomical data, including orbital volume and RBH volume were performed using the Brainlab Server. The orbital volume and RBH volume were compared with each other (orbit-to-RBH ratio) in each patient.

The data processing and statistical evaluation were performed with SPSS ver. 29.0 (IBM, USA) and Microsoft Excel 2021 (Microsoft Corp., USA). Analyses included descriptive statistics, Kolmogorov-Smirnov-test, χ2-test for group comparisons and t-test for comparison (level of significance: *p* < 0.05).

Neural networks have already been used in analyzing risk factors that lead to cleft palate [45] and even to assess need for surgery in traumatic brain injuries [46]. We used the standard multilayer perceptron setup of the SPSS to perform the risk analysis.

To further analyze the impact of the different factors, a receiver operating characteristic (ROC) analysis was conducted to evaluate the discriminative performance of the RBH-to-orbit volume.

A multiple linear regression model was fitted to predict the outcome using the following predictors:

- Demographics: age and gender.
- Clinical/anatomical variables: anticoagulation, fracture zone, hematoma localization related to the muscle cone (extra- or intraconal), contact to optic nerve and contact to globe, as well as.
- Quantitative measure: RBH-to-orbit ratio.

All predictors were entered simultaneously (ENTER method).

The results are presented in the following tables and figures (Table 1).

## Results

**Table 1:** The table repesents the most important demographic data for the study

Sex	Total study population		Patients with RBH	
	*N*	Average age (years)	*N*	Average age (years)
Female	2894	53.9 ± 32.2	24	71.1 ± 19.5
Male	4777	41.7 ± 26.4	28	48.1 ± 23.6
Total	7671	46.3 ± 29.3	52	58.7 ± 24.5

(N=Number of patients in a specific age group)

**Fig. 2:**
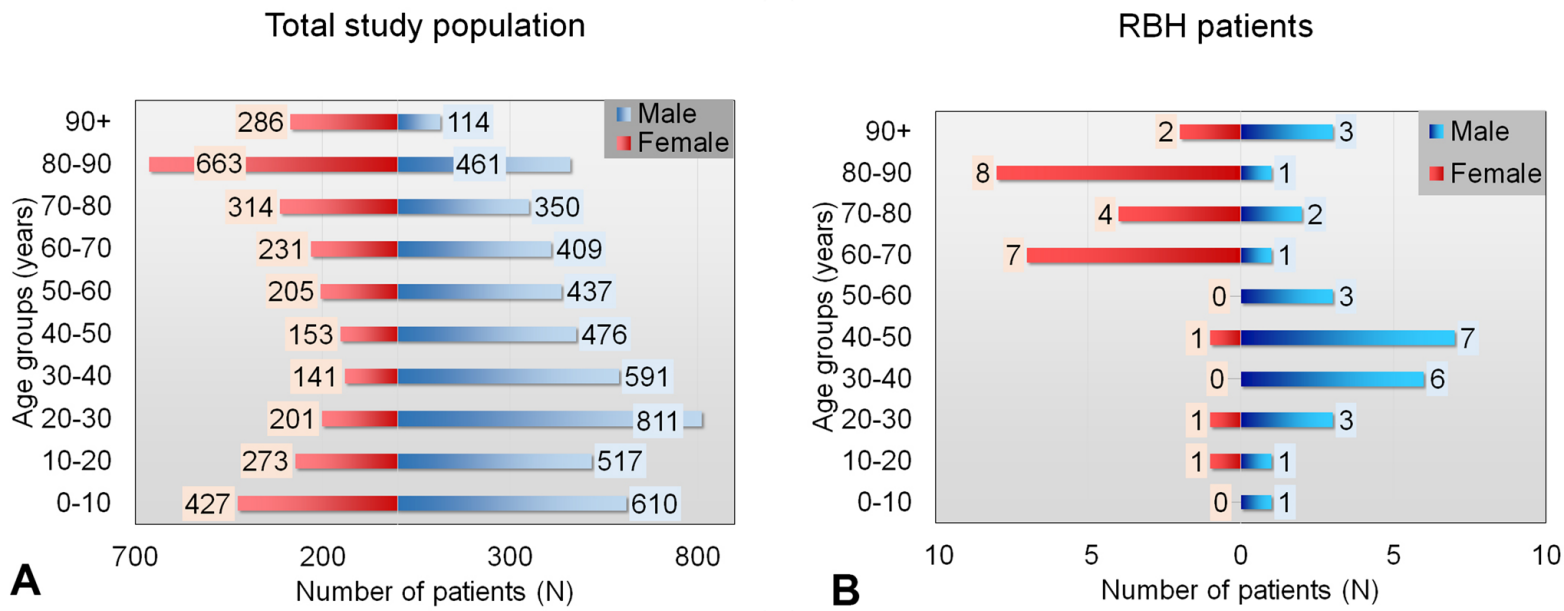
Sex and age differences: Diagram **A** presents the age distribution of the total study population (all trauma cases); Diagram** B** presents the age distribution of patients with RBH

The Dortmund Maxillofacial Trauma Registry (DMTR) includes 7671 patients with head and neck trauma from 1.1.2018 to 31.3.2025. 52 patients (0.7%) presented with RBH (28 of them were males and 24 females), resulting in a male-to-female ratio of 1.2:1. The diagrams of the Fig. 2 show a different trend in the two genders. In females, traumatic injuries had a relatively low incidence after childhood. In elderly women (aged 60–90 years), the incidence of traumatic injuries and RBH both increased. RBH is scarcely found in females before the age of 60. Contrarily, RBH occured across all age groups in males, including in young children, peaking in the age groups of 30–40 and 40–50. Middle-aged males and elderly females appeared at greatest risk.

The analysis of pre-existing medical conditions (Table 2) identified hypertension, diabetes mellitus and other endocrine disorders as the most abundant concomitant diseases in the RBH group. In the overall study population hypertension, neurological disorders and endocrine disorders were the leading conditions.

**Table 2:** Distribution of pre-existing medical conditions in the total study population and in the RBH group

	RBH subgroup		Total study population	
	*N*	Rank	*N*	Rank
Hypertension	23	1	2016	1
Diabetes mellitus	8	2	633	5
Endocrine disorders	7	3	740	3
Neurological disorders	6	4	749	2
Tumour	5	5	233	8
Atherosclerosis	3	6	740	3
Eye disease	2	7	227	9
Dementia	2	7	539	6
Bleeding disorder	1	9	52	13
Acute myocarditis	1	9	200	11
Valvular heart disease	1	9	235	7
Stomach diseases	1	9	225	10
Hematological disorder	0	13	164	12
Other	28		2728	

The ranking of the disease groups was performed based on the RBH group. Patients can appear in more than one medical condition

The most important medications that can lead to excessive bleeding are anticoagulants (Table 3): The use of these medications was 19–38 times more frequent in the RBH subgroup compared to the total study population. In the RBH group, 8 patients took 100 mg aspirin daily, 5 were on novel anticoagulants and 2 on vitamin K antagonists. (Other type of thrombocyte aggregation inhibitors or heparine derivates were not used by RBH patients and were rare in the overall population.)

**Table 3:** Comparison of the concomitant medications in the RBH and total study group. The % data for both groups is calculated based on the total numbers provided in Table 1. The comparison of the usage intensity in the RBH group was based on the % data

RBH	RBH subgroup		Total study population			X-fold RBH to Total
	%	Rank	N	%	Rank	
Aspirin	15.40%	1	27	0.50%	2	30.8x
NOAC	9.60%	2	24	0.30%	3	32x
Marcumar	3.80%	3	5	0.10%	4	38x
Antiresorptives	3.80%	3	2	0.20%	6	19x
Beta-blocking	0.00%	4	46	0.00%	1	-
Thrombocytes aggregation	0.00%	4	4	0.00%	5	-

The % data for both groups is calculated based on the total numbers provided in Table 1. The comparison of the usage intensity in the RBH group was based on the % data

**Fig. 3:**
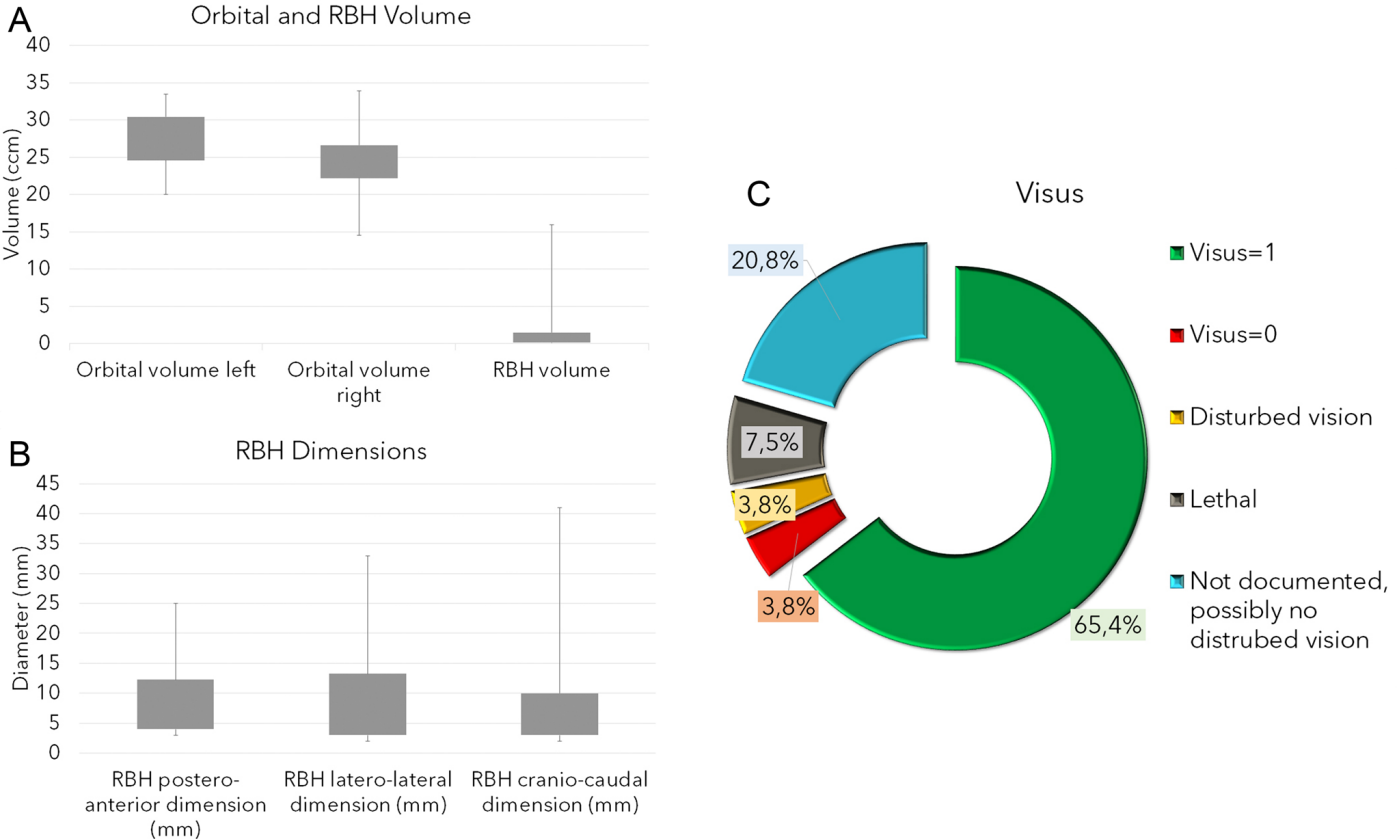
Characterization of RBH data. (**A**) Representing the maximal dimensions of the RBH in millimetres (postero-anterior, latero-lateral and cranio-caudal length); (**B**) Comparison of the orbital volumes in cm^3^ on both sides and the RBH volume (in cases of bilateral RBH, the average was taken into consideration); (**C**) Outcome analysis: Visus = 1 is maximal sight reported, Visus = 0 no eyesight

The orbit and RBH were further examined in all patients with RBH (Fig. 3). The average extension of the RBH were 14.1 ± 8.1 mm in the postero-anterior, 13.3 ± 8.3 mm in the latero-lateral and 13 ± 11.1 mm in the cranio-caudal dimension. The average orbital volume in 52 patients was 31.9 ± 3.8cm^3^ on the left side, while 32.5 ± 4.6cm^3^.on the right side. The RBH-to-orbital volume ratio was 2.9 ± 0.5 on the left side (> 1 also indicating extraorbital extension) and 0.01 ± 0.02 on the right side.

64.2% (*n* = 34) of RBH patients were dismissed from hospital with intact vision. Further 20.8% (*n* = 11) had no explicit visus data for the discharge day. 3.8% (*n* = 2) had disturbed vision and further 3.8% (*n* = 2) had no vision at all. A total of 7.8% (*n* = 4) died in hospital due to the severity of the trauma or other organ failure.

**Fig. 4:**
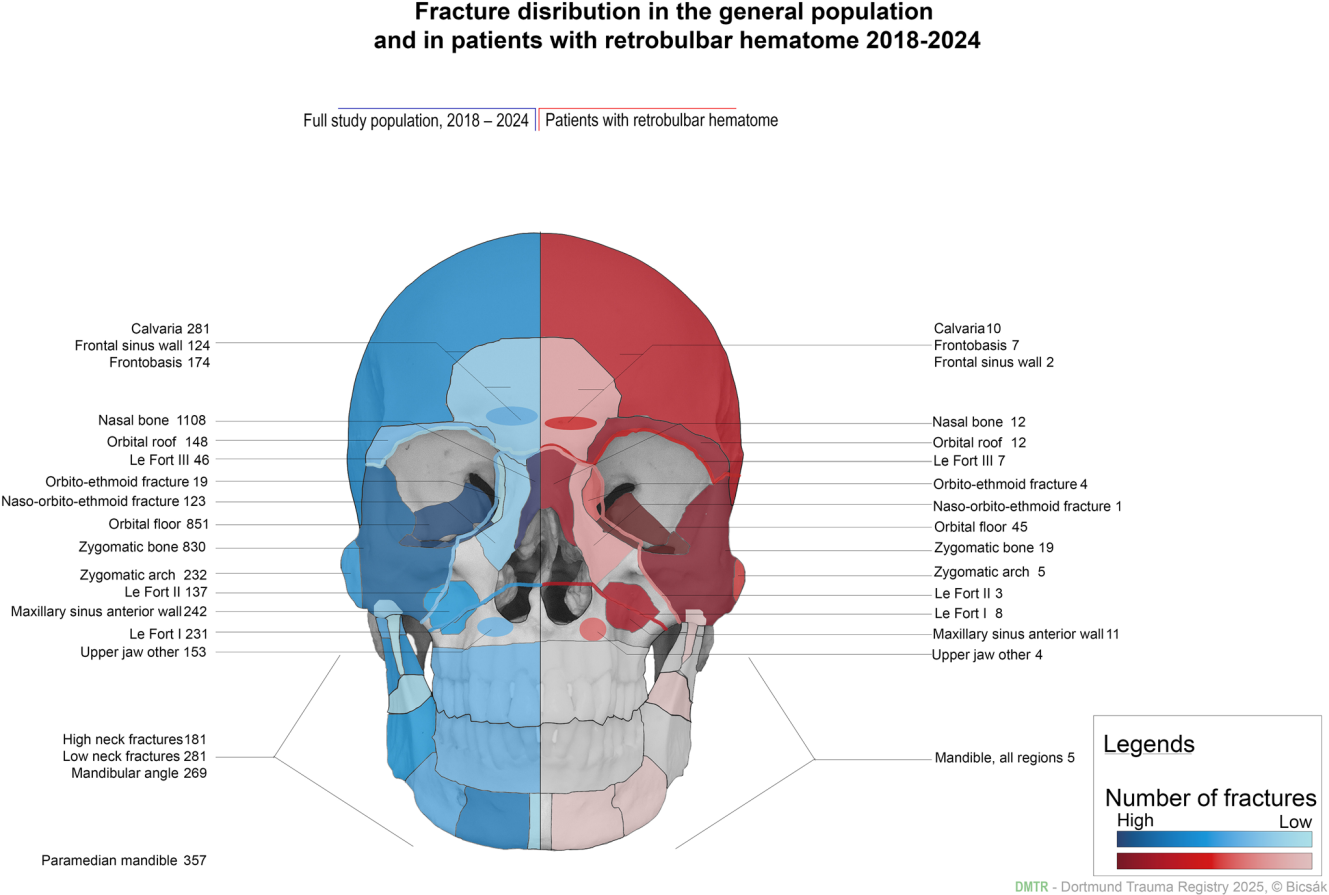
Heatmap representing the fracture distribution of all 7671 patients with head and neck injuries (total study population; left) and the fracture distribution in RBH patients (right). Please note that patients can have several fracture sites concomitantly (numbers are provided according to anatomical locations)

The diagnosed fracture sites are presented in a heatmap in Fig. 4. Fractures sites of the total study population (left side of the skull, Fig. 4), are predominantly located at the nasal, frontal and zygomatic bones, orbital floor fractures and also at the mandible (mandibular condyle, angle and paramedian). Contrarily, in RBH patients fracture sites are predominantly located around the orbit (right side of the skull, Fig. 4): Fractures of the basal bone, the orbital floor, the zygomatic bone, the anterior maxillary sinus wall, the orbital roof and frontal bone are most common (Frankfurt horizontal plane). As opposed to the fracture localizations of the total study population, fractures in the mandible or below the Le Fort-I plane are much less common in RBH patients.

**Fig. 5:**
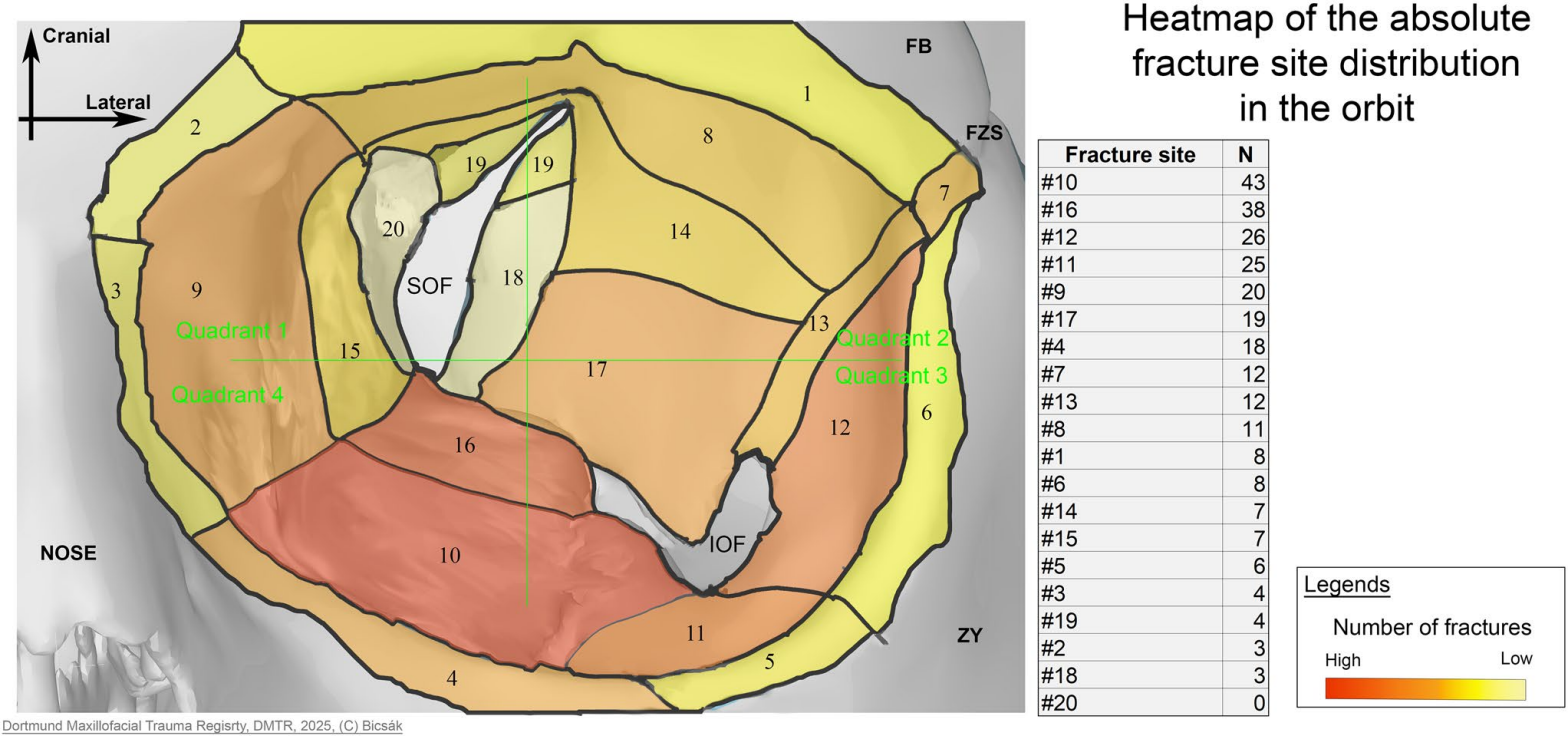
Heatmap of the distribution of the intraorbital fracture sites in the RBH group (*n* = 52). The arabic numbers in the different areas represent the fracture site localization regarding the AO level 3 classification [47]. The colours represent the relative incidence of the fractures in each of these regions. The number of fractures in each of these areas are listed in the table on the right hand side of the figure FB = frontal bone; FZS = fronto-zygomatic suture; IOF = inferior orbital fissure; SOF = superior orbital fissure; NOSE = nasal bone; ZY = zygomatic bone

A detailed analysis of the intraorbital fracture sites in the RBH group is based on the Level 3 AO classification of orbital fractures [47] and is presented in Fig. 5. Predominant fracture sites were the orbital floor posterior to the orbital rim (Zone 10), the orbital floor dorsally (Zone 16), the lateral orbital floor and the orbital surface of the zygomatic bone (Zones 11 and 12) as well as the medial orbital wall (Zone 9, lamina papyracea of the ethmoid). These five zones represent 55.5% of all fracture sites, together with zones 17 and 4 even 69% of all fracture sites. The intra-orbital fractures associated with RBH also align to the Frankfurt horizontal plane / orbital floor.

The analysis of the hematoma position showed that 19 RHBs were located intraconally, 30 extraconally, 2 extra- and intraconally and 2 extraconally with periorbital hematomas (including one bilateral case). Thirteen RHBs were in contact with the optical nerve, and 7 with the bulb. Hematomas were located in all quadrants: 15/52 (28.8%) in quadrant 1, 22/52 (42.3%) in quadrant 2; 14/52 (26.9%) in quadrant 3 and 20/52 (38.5%) in quadrant 4.

Table 4 lists the different treatments that were chosen. Of 52 patients, 31 were treated conservatively. This often happened when the onset of disturbed vision manifested only after a prolonged time span after the injury or in case of missing symptoms when a RBH was only accidentally detected. In 10 cases, a combined lateral canthotomy and infraorbital decompression was performed, in 6 cases an infraorbital decompression was performed, one case underwent only lateral decompression, and in 4 cases a different surgical approach (for example through an existing soft tissue injury) was used.

**Table 4:** Summary of chosen surgical treatment

Treatment	*N*
Lateral decompression	1
Infraorbital decompression	6
Lateral and infraorbital decompression	10
Other surgery	4
Conservative	31
Total	52

**Fig. 6:**
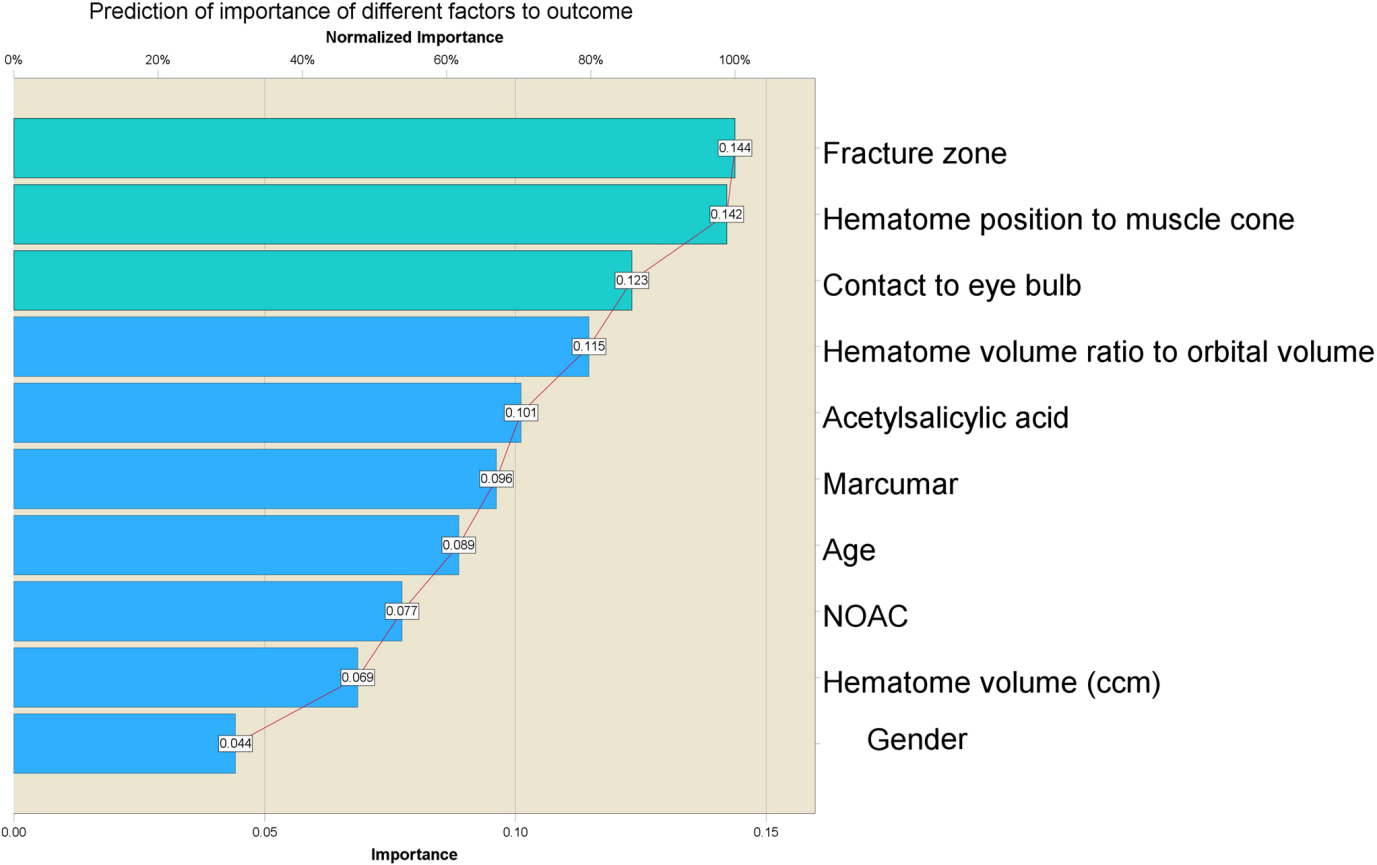
Analysis of the effective weight of different factors affecting the outcome performed by neural network (multilayer perceptron method). The highest weighted bars are marked with different colour for better understanding. The red line and associated values express relative weight of each factor

In further analysis, a neuronal network (SPSS, multilayer perceptron) was trained with the data to assess the effect of the different analyzed factors regarding the outcome (visus at the time point of the dismission from hospital) of the RBH treatment (Fig. 6). This analysis found the orbital floor fracture zone (see in the heat-map), the intraconal hematoma location, contact of the RBH with the globe, a high RBH-to-orbital volume ratio, and acetylsalicylic acid usage (or summarized anticoagulation/anti-platelet therapy) to be predictive factors for a bad outcome. Other concomitant medication, age, gender, absolute RBH volume, absolute hematoma dimensions or concomitant diseases were not significant predictors. Anticoagulation therapy data is acquired together with the medical history. All other important parameters can easily be assessed in CT-scans.

The receiver operating characteristic (ROC) analysis was conducted to evaluate the discriminative performance of the hematoma-to-orbit ratio for predicting the outcome variable. The analysis included 53 evaluable cases (2 positive, 51 negative), with 2 cases excluded due to missing data.

The area under the ROC curve (AUC) was 0.824 (standard error = 0.084, 95% CI: 0.659–0.988, *p* < 0.001), indicating good overall discriminatory ability of the hematoma ratio for distinguishing between outcome groups. The lower bound of the confidence interval remains well above 0.5, further supporting statistically significant predictive performance.

Classifier performance metrics demonstrated a Gini index of 0.647, consistent with the AUC estimate (Gini = 2×AUC − 1), and a maximum Kolmogorov–Smirnov (K–S) statistic of 0.725, suggesting strong separation between the distributions of positive and negative cases.

The optimal cutoff value, based on the maximum K–S statistic (equivalent to maximizing Youden’s index), was identified at approximately 0.0544. At this threshold, the model achieved: Sensitivity: 1.00 (100%) and Specificity: approximately 72.5% (1 − 0.275).

Therefore, for clinical use, we can suggest a 0.05 (5%) RBH-volume to total orbit volume as threshold for impaired visual acuity.

Inspection of the ROC coordinates revealed that sensitivity remained at 100% across a broad range of low cutoff values, reflecting the model’s ability to correctly identify all positive cases, albeit at the cost of reduced specificity at lower thresholds.

### Multivariable regression analysis

For the multivariate analysis, the patients with outcome “lethal” were excluded, the residual outcome was converted into binary model (“known impairment of visual acuity” and “no known impairment”). 49 patient data was included in the analysis. The overall regression model was not statistically significant (F(8,40) = 1.398, *p* = 0.227), demonstrating limited explanatory power (R² = 0.219; adjusted R² = 0.062). The standard error of the estimate was 0.268, indicating modest dispersion of residuals around the fitted values.

Among the included predictors, contact with the nerve was the only variable significantly associated with the outcome (B = − 0.288, SE = 0.119, β = −0.452, *p* = 0.020). The negative coefficient indicates that nerve contact is associated with a reduction in the outcome measure. The 95% confidence interval (− 0.528 to − 0.047) did not cross zero, supporting the robustness of this association.

Anticoagulation (*p* = 0.245), relation to the muscle cone (*p* = 0.269), contact with the eyebulb (*p* = 0.288) showed a non-significant, but marked association to the outcome. All other variables were not statistically significant, including age (*p* = 0.631), gender (*p* = 0.942), zone classification (*p* = 0.654), and hematoma ratio (*p* = 0.868).

### Correlation analysis

Bivariate analysis demonstrated a moderate negative correlation between outcome and nerve contact (*r* = − 0.350, *p* = 0.007), consistent with the multivariable findings. Other correlations with the outcome were weak and did not reach statistical significance.

### Collinearity diagnostics

Assessment of multicollinearity revealed no significant concerns, with variance inflation factors ranging from 1.13 to 1.79 and tolerance values above 0.5 for all predictors. Condition indices were within acceptable limits, indicating that collinearity did not materially influence the regression estimates.

### Residual analysis

**Fig. 7:**
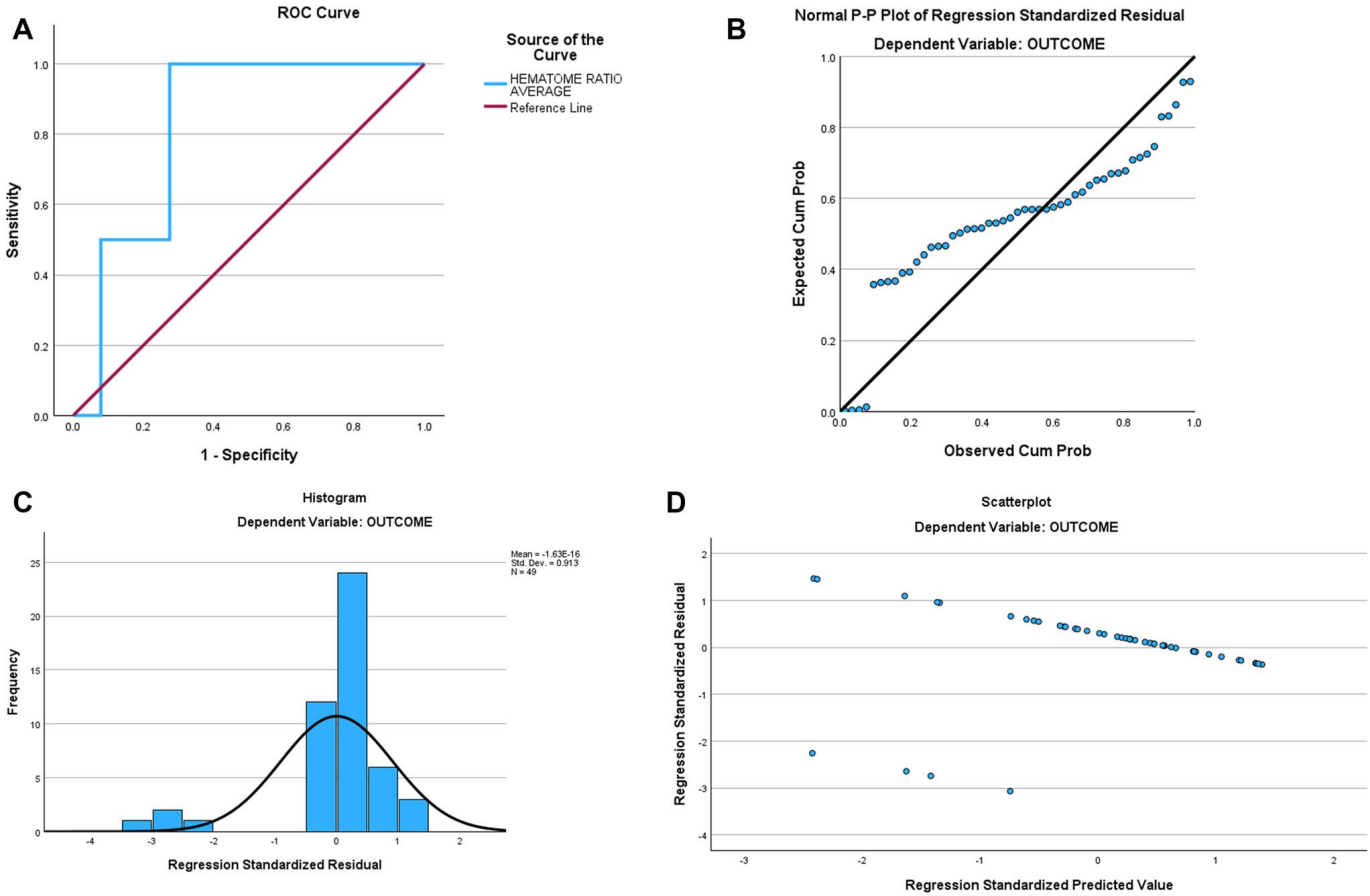
(**A**) receiver operating characteristic (ROC) analysis. (B-D) Presentation of the mulitvariable linear regression analysis

Residual diagnostics showed a mean residual of zero with a standard deviation of 0.245. Standardized residuals ranged from − 3.07 to 1.47, suggesting the presence of at least one potential outlier, although no systematic deviation from model assumptions was observed (Fig 7)

## Discussion

Big data analysis allows us to identify potential factors leading to poor outcomes as well as determinants that can improve clinical outcomes. We also utilized “digital twins” [48], segmentation and a three-dimensional work-flow to improve anatomical insights while obtaining the most accurate measurements.

In our cohort of 7671 patients with midface fractures, the incidence of retrobulbar hematoma (RBH) was 0.7% (*n* = 52), aligning with the 0.7% reported by Blumer et al. [49] Most cases were traumatic, postoperative RBHs were rare. Females in both the general study population and the RHB subgroup were older than males, with a more pronounced age gap in the RHB group. The mean age of RBH patients was comparable to most published series [15, 18, 27, 49, 50]. Except in India, children appear at greatest risk [51]. Falls were the leading cause, followed by interpersonal violence and bicycle accidents [49]. Orbital floor fractures and zygomatic bone fractures predominated. Reported RBHs rates range from 0.16% (post-operative only) [1] or in traumatic settings 0.3% to 31% [16, 24, 25, 27–29, 49, 52]. Bilateral cases are rare – only 1/52 in our sample which is consistent with previous reports [28, 52, 53]. While our male-to-female ratio was balanced, Ericson and Garcia reported 80% male predominance [16].

Visual prognosis was favourable in nearly two-thirds of the patients: 64.5% recovered full visual acuity, similar to the reported rates of 40%-62.5% [5, 29, 52, 54], in 94% of partly post-operative RBHs [26] and 100% in children [51]. Blindness occurred in 3.8%, which is lower than the 7.4%-60% that were reported internationally [5, 29, 52, 54]. Outcome data was incomplete for 20.8% of our patients, suggesting our recovery rates may be underestimated. Lethality was 7.8%, likely reflecting the severity of concomitant craniofacial and systemic injuries.

Key predictors of blindness in our series were the fracture localization (orbital floor), higher hematoma-to-orbit ratio, and contact of the RBH with the globe. Age, anticoagulation and other factors were less influential. In comparison, Christie et al. identified symptom onset time, impact zone, and direct trauma as most important factors [29], while Dixon et al. [27] emphasized intraocular pressure measurement as the best predictor. Given that lateral canthotomy can reduce intraorbital pressure (IOP) by 50% [16], immediate decompression should be performed in all cases where IOP elevation is suspected.

Anticoagulant and antiplatelet therapy increased the bleeding risk: 15 of 52 (28.8%) of our RBH patients were on such medication, compared to 36% [17] and 50% [18] in prior studies and compared to 0.7% (52 of 7671) of the total study population. In Maurer et al.’s series [55] of 68 anticoagulated elderly patients, RBH occurred in 6 (8.8%), with only one regaining visual acuity.

Mean orbital volume was 31-32mm^3^, slightly larger than the 24.5mm^3^ reported by Andrades et al. [56], likely due to methodological differences in segmentation techniques and software. Mean hematoma volume was 3.2 ± 10.5mm^3^ in average, approximately 1% of the orbital volume (excluding one extra-intra-orbital hematoma with a huge extraorbital part). To our knowledge, no other study reports such volumetric data. Riekert et al. [15] used digital twin analysis but provided no absolute or relative volume values, though they confirmed a correlation between hematoma size and blindness. Despite frequent CT detection of RBH, no prior study has yet determined its predictive value for OCS or severe disability [28].

We also conducted the first detailed mapping of orbital fracture sites associated with RBH using the AO level 3 classification [47]. Fractures most frequently aligned with the Frankfurt horizontal plane, consistent with our prior finding that facial fractures associated with cervical spine injuries follow the same alignment [57]. This underscores the biomechanical significance of the Frankfurt horizontal plane and merits further investigations possibly with digital twin modelling- to enhance understanding of concomitant craniofacial injuries and e.g. improve industry standards for prevention.

Management followed a conservative or surgical pathway. Conservative treatment (500 mg – 1000 mg methyl-predinisiolone iv., repeated within 24 h, further dosing individualized) was reserved for patients with high operative risk or symptom onset 24 h after the incident, consistent with the literature [7].

Surgical intervention -within 24 h of the incident- included lateral canthotomy [2, 5, 7, 14, 25, 58, 59] – often combined with an infraorbital approach [58] and temporary soft rubber drainage (for 2–3 days). We avoid the transconjunctival approach [58] in favour of subtarsal incision for optimal drain positioning and wound observation. An antibiotic coverage consisted of ampicillin – sulbactam 3 g iv. three times a day.

Multilayer perceptron analysis identified intraconal location, contact with the globe or optic nerve, and relative RBH size as the strongest predictors for outcome. Although we lacked sufficient data on time to decompression, our clinical impression supports minimizing this interval to optimize prognosis.

The ROC analysis demonstrates that the RBH-to-orbit volume ratio is a robust discriminator for the studied outcome, with an area under the curve of 0.824 indicating good diagnostic accuracy. A key finding is the 100% sensitivity at the optimal cutoff. However, this comes with a trade-off in specificity (~ 72.5%), implying a moderate rate of false positives.

The high K–S statistic (0.725) further supports strong class separation, suggesting that the distribution of hematoma ratio values differs substantially between outcome groups. This strengthens the argument that the variable captures meaningful underlying pathophysiological differences. Thus the RBH-to-orbit volume ratio shows promising discriminatory performance, but the findings should be interpreted cautiously due to the limited number of positive cases. Future studies with larger and more balanced datasets are necessary to validate the robustness of these results and to refine clinically applicable cutoff thresholds.

The regression model demonstrated modest explanatory capacity (R² = 0.219) with a substantial reduction after adjustment (adjusted R² = 0.062), highlighting the impact of model complexity relative to sample size. The results indicate, that predictors of a poor outcome are processes that have an impact on the localization of the intraorbital presence of hemorrhage. Age, gender and other external factors have no influence. We have to admit that the optimal sample size for this regression model is 2.5-3x higher. Therefore, taking clinical experience in consideration, we accept a marked association also as a clinically important factor.

In summary, RBH after midface fracture remains rare but potentially vision-threatening. Early recognition, attention to volumetric parameters, and prompt decompression when indicated are critical. Our findings support a structured checklist approach (Table 5): the greater the number of positive risk indicators, the higher the likelihood of permanent vision loss. The checklist is short, summarizes clinical and radiological symptoms and the duration of the symptoms, is an easy-to-use tool even in emergency room conditions. The usage does not contain any speciality related expressions, the assessment can also be performed by trained emergency room staff supporting quick medical decisions.

**Table 5:** RBH checklist to assess the risk of loss of visual acuity

Factor	Present?
Anticoagulants?	
Big RBH related to the orbital volume?	
Contact to nerve / bulb?	
Intraconal RBH?	
Orbital floor fracture?	
Sudden-onset, extreme orbital pain	
Time passed since symptom onset >1 h	
Time passed since symptom onset >2 h	

### Limitations of our study

This was a monocentric, retrospective study. While our maxillofacial traumatology database has been shown to represent case distributions across Germany, Austria, and Switzerland [30], this has not been specifically validated for RBH. Nevertheless, the close agreement of our results with an independent Swiss report [49] suggests good external comparability.

The retrospective design reflects both necessity and ethical constraints: any study design deviating from the best-known treatment for RBH would unacceptably risk permanent disability. In this context, we consider a well-conducted retrospective analysis to provide the highest feasible level of evidence for RBH-related questions.

Data on intraocular pressure were unavailable, as measurement in emergency settings is often considered unreliable [27]. In our view, clinical symptoms alone are sufficient to define RBH and OCS and to trigger urgent surgical intervention.

Precise documentation of the symptom onset–to–surgery interval was often lacking, precluding robust analysis. This omission reflects the clinical reality that, in acute RBH, the attending surgeon’s priority is immediate intervention, not simultaneous record-keeping. Symptom onset is often in the prehospital phase of the trauma care process, also often before the arrival of the ambulance at the accident site. In a real emergency room situation, the work load is extreme, in most centres, only one maxillofacial surgeon is present. We support acting without delay and documenting the procedure after all necessary tasks have been done. Due to differences in the documentation requirements of a study and emergency care, missing data must be accepted but reduced as much as possible.

Both the ROC analysis and the multivariate analysis showed acceptable results, however, the optimal number of cases would be approximately 2-2.5x more cases. This indicates a necessity of international multicenter studies. The results of these could provide more data on relevant predictors of poor outcome.

## Conclusion

We recommend performing an ophthalmologic examination in all injured patients with midface fractures, as well as in patients without a fracture presenting with periorbital hematomas at the earliest possible time point. This is essential to promptly identify post-traumatic orbital or ocular hematomas, retinal detachment, traumatic iritis, optic nerve avulsion, lens dislocation and corneal injuries [3]. Early detection and timely and adequate treatment of these conditions can preserve patients’ visual capacity [22].

Surgical decompression should be performed immediately if any symptoms present. In our experience, even colleagues in remote emergency departments without prior canthotomy experience can be successfully guided to perform the procedure, if the transport duration would exceed the critical intervention window.

As delayed bleeding – and thus a later onset of hemorrhage formation – is possible, we recommend inpatient observation in line with current literature [5, 25, 60]. The observation should be performed by trained personal and must include a detailed ophthalmological examination. We suggest a minimum observation period of 48 h, as some literature reported worsening of the visual acuity even after 24 h [60].

If the CT scan is the first to describe the RBH -as may occur in unconscious, ventilated, cognitively impaired patients, or in asymptomatic RBH immediate orbital re-evaluation is mandatory.

## Data Availability

Data availability is governed by the Data Protection laws of the European Union, the Federal Republic of Germany, the State of North Rhine-Westphalia, and the regulations of Dortmund General Hospital. Data is, therefore, purposely made available on-site.

## Abbreviations

AO: AO (Allgemeine Osteosynthesegruppe) Foundation
AOCS: Acute orbital compartment syndrome
CT: Computed tomography
IPV: Interpersonal violence
iv: Intravenous
mg: Milligram(s)
OCS: Orbital compartment syndrome
PLR: Deficient pupillary light reflex
RAPD: Reactive afferent pupillary defect
RBH: Retrobulbar hematoma
ROC: Receiver operating characteristic analysis
